# Human Papillomavirus Vaccine Uptake among Individuals with Systemic Inflammatory Diseases

**DOI:** 10.1371/journal.pone.0117620

**Published:** 2015-02-18

**Authors:** Candace H. Feldman, Linda T. Hiraki, Huichuan Lii, John D. Seeger, Seoyoung C. Kim

**Affiliations:** 1 Division of Rheumatology, Immunology and Allergy, Department of Medicine, Brigham and Women’s Hospital, Boston, Massachusetts, United States of America; 2 Division of Rheumatology, The Hospital for Sick Children, Toronto, Canada; 3 Division of Pharmacoepidemiology and Pharmacoeconomics, Brigham and Women’s Hospital, Boston, Massachusetts, United States of America; The Ohio State University, UNITED STATES

## Abstract

**Objectives:**

The human papillomavirus (HPV) vaccine is safe and efficacious in patients with systemic inflammatory diseases (SID) who have higher rates of persistent HPV infection. We compared HPV vaccine uptake among SID and non-SID patients.

**Methods:**

Using a U.S. insurance claims database (2006–2012), we identified individuals 9–26 years with ≥2 SID diagnosis codes ≥7 days apart with ≥12 months of continuous enrollment prior to the second code (index date). We matched SID patients by age, sex and index date to randomly selected non-SID subjects and selected those with ≥24 months of post-index date continuous follow-up. We also identified a non-SID subcohort with ≥1 diagnosis code for asthma. We defined initiation as ≥1 HPV vaccination claim after 2007, and completion as 3 claims. We used multivariable logistic regression to assess uptake in females 11–26 years comparing SID, non-SID and asthma cohorts, adjusting for demographics, region, comorbidities, and healthcare utilization.

**Results:**

We identified 5,642 patients 9–26 years with SID and 20,643 without. The mean age was 18.1 years (SD 4.9). We identified 1,083 patients with asthma; the mean age was 17.2 (SD 5.1). Among females, 20.6% with SID, 23.1% without SID and 22.9% with asthma, received ≥1 HPV vaccine. In our adjusted models, the odds of receipt of ≥1 vaccine was 0.87 times lower in SID (95% CI 0.77–0.98) compared to non-SID and did not differ for 3 vaccines (OR 1.03, 95% CI 0.83–1.26). The odds of initiation and completion were not statistically different between SID and non-SID asthma cohorts.

**Conclusions:**

In this nationwide cohort, HPV vaccine uptake was extremely low. Despite the heightened risk of persistent HPV infection among those with SID, no increase in HPV vaccine uptake was observed. Public health efforts to promote HPV vaccination overall are needed, and may be particularly beneficial for those at higher risk.

## Introduction

Human papillomavirus (HPV) is the most common sexually transmitted disease (STD) in the U.S. and a cause of cervical, vaginal, vulvar, penile, anal and oropharyngeal cancers.[1] Individuals with systemic inflammatory diseases (SID), specifically autoimmune diseases including inflammatory bowel disease (IBD), systemic lupus erythematosus (SLE), and possibly rheumatoid arthritis, have an increased risk of persistent HPV infection and cervical cancer.[2–4] Studies also suggest increased rates of cervical intraepithelial neoplasia among patients with SLE and IBD.[5,6] This may be due both to the nature of SID and to the immunosuppressive therapies used for treatment.

In 2006, the U.S. Food and Drug Administration approved a quadrivalent HPV vaccine that protects against HPV types 6, 11, 16 and 18, for females aged 9–26 years. In 2007, the U.S. Advisory Committee on Immunization Practices (ACIP) recommended use among females aged 9–26 years, and specifically, routine use among females aged 11–12, and among 13–26 year-olds if they were not vaccinated earlier.[7] In 2009, the quadrivalent vaccine was approved for use in males 9–26 years, and the ACIP encouraged use for prevention of genital warts however, routine use was not recommended until 2011. A bivalent vaccine against HPV types 16 and 18 was also approved for females 10–25 years in 2009, and for 9 year-olds in 2011. Despite substantial evidence of vaccine efficacy, and no serious safety concerns, vaccine uptake in the U.S. has lagged behind other recommended vaccines, and behind other parts of the world.[8] This lag has been attributed to inconsistent provider recommendations, absence of state and school-wide mandates for administration, insufficient parental education, and sensitivity surrounding conversations about sexual activity and STDs.[8–11]

A few studies have investigated the safety and efficacy of the HPV vaccine among individuals with SID. Adverse reactions have been minimal and no increase in SID flares occurred.[12] One study demonstrated somewhat lower seroconversion rates in SLE patients, however the vaccine was deemed reasonably efficacious in this population.[13] While several case reports hypothesize a relationship between autoimmune disease incidence and HPV vaccination,[14] two large, observational studies demonstrate no increased risk of autoimmune disease among vaccinated women.[15,16]

We investigated HPV vaccine uptake among individuals with SID compared with those without SID, and to a subset without SID with asthma, the most common chronic disease in U.S. children. [17] We limited our definition of SID to autoimmune diseases that are treated with immunosuppressive medications since the associated increased risk of persistent HPV infection and cervical cancer has been shown in a number of studies to be higher in this group.[4,6,18,19] Given this increased risk, we hypothesized that higher vaccine uptake may be observed among individuals with SID compared to the general population with and without other chronic diseases.

## Methods

**Fig 1:**
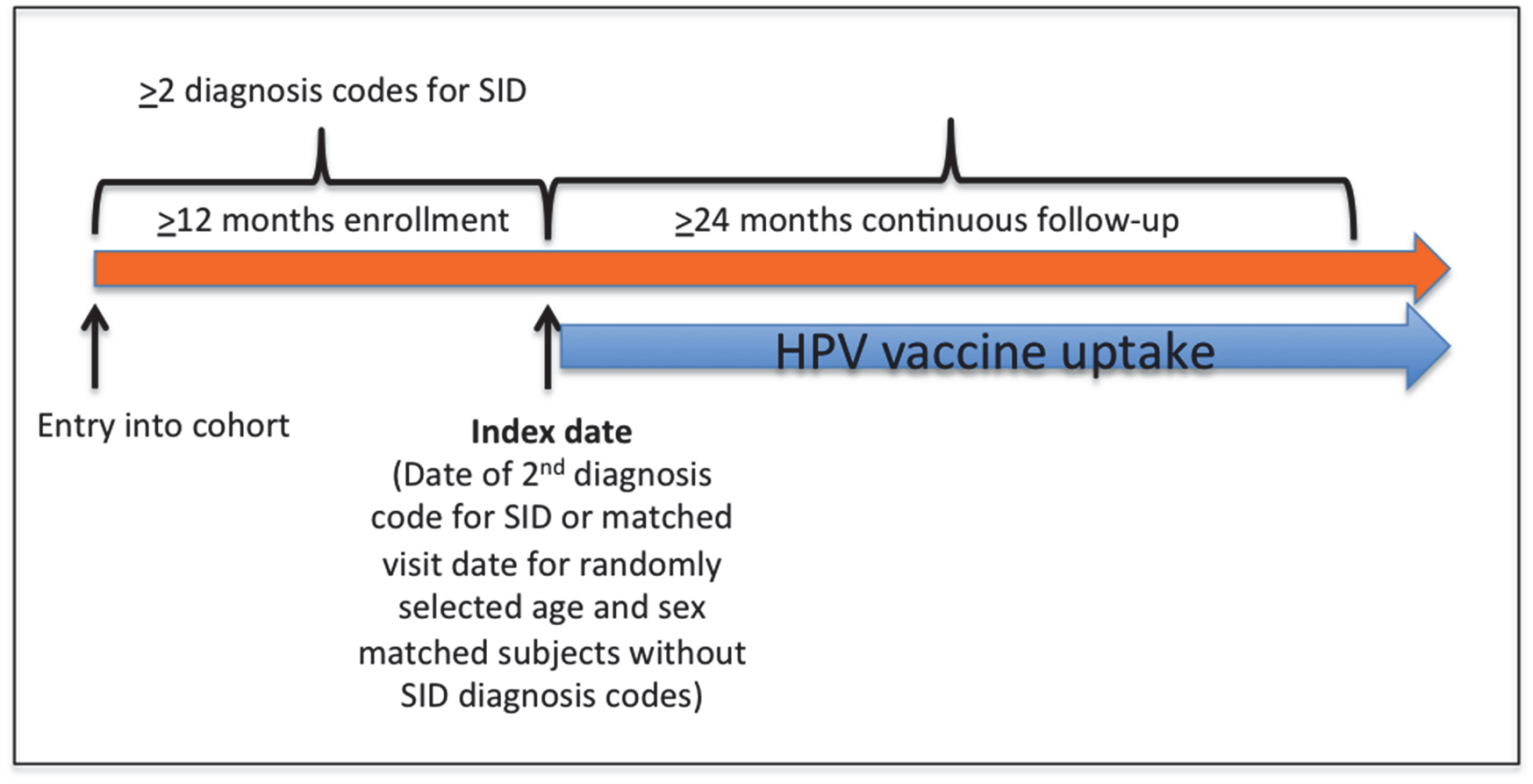
Overview of Study Design. Among subjects with ≥12 months of continuous enrollment, we identified those with ≥2 diagnosis codes for SID during this time period. The index date was defined as the date of the second diagnosis code (SID) or visit date (non-SID). SID and non-SID subjects were matched by age, sex and index date (1:4 ratio). In SID and non-SID matched subjects, we assessed HPV vaccine uptake beginning in 2007 among those with ≥24 months of continuous follow-up.

We used claims data from United HealthCare, a nationwide U.S. commercial insurance that covers primarily working adults and their families from January 1, 2006 through March 31, 2012. We identified patients aged 9–26 years with ≥2 SID diagnosis codes ≥7 days apart and ≥12 months of continuous enrollment prior to the second code (Fig. 1). SID included: juvenile idiopathic arthritis, rheumatoid arthritis, SLE, psoriasis, psoriatic arthritis, IBD, vasculitis, multiple sclerosis, juvenile dermatomyositis, systemic sclerosis, ankylosing spondylitis, Goodpasture’s syndrome, and sarcoidosis. We included SID for which immunosuppressive therapy is used due to the heightened likelihood of persistent HPV infection and the potential increased risks of cervical cancer documented in this group. Therefore, we did not include autoimmune diseases such as celiac disease and autoimmune thyroiditis because immunosuppressive medications are not used and because an increased risk of cervical cancer has not been reported. Based on the findings of one study that suggested a possible modest increase in the risk of cervical cancer among patients with diabetes mellitus type 1 (DM1), we separately examined HPV vaccine uptake in a small subset with this diagnosis.[20] To identify patients with DM1, we utilized ≥1 ICD-9 code for DM1 (PPV 95.5%) during our baseline period.[21]

We defined the index date as the calendar month and year of the second SID diagnosis code. We matched these individuals by age and sex to randomly selected subjects with ≥12 months of continuous enrollment who had a visit for a non-SID diagnosis at the same index date without any SID diagnosis codes (1:4 ratio). We also identified a non-SID subset of patients with asthma, the most common chronic disease among children in the U.S., to compare uptake to the SID population. [17] We defined asthma cases using ≥1 ICD-9 code based on a prior study demonstrating the adequacy of this method for research purposes.[22] We excluded individuals with malignancy, organ transplantation, or less than 24 months of continuous follow-up after the index date.

Beginning in 2007, we defined the primary outcome as HPV vaccine initiation: ≥1 HPV vaccine code (CPT 90649, 90650) among individuals with ≥24 months of continuous follow-up after the index date to allow for adequate time enrolled in the health plan to complete the vaccine series. Given the ACIP recommendation for use in females in 2007 and full coverage by the United HealthCare plan in 2007, we used the time period between 2006–2007 only to determine SID versus non-SID status and did not begin follow-up to assess vaccine uptake until 2007. We also evaluated vaccine completion (3 vaccine codes). Individuals were censored at the completion of the HPV vaccine series, the end of study period, or disenrollment. To determine whether length of enrollment in the health plan contributed to increased HPV vaccine uptake, as a sensitivity analysis, we assessed individuals with ≥12 months and ≥36 months of continuous follow-up after the index date.[22] In males, we documented vaccine uptake but did not conduct further analyses given the recent dates of approval and the limited time frame of our available data. [22]

Baseline covariates included age, sex, U.S. geographic region, healthcare utilization (outpatient visits, specifically to primary care physicians and obstetrics/gynecology), and comorbidities (smoking, sexually transmitted diseases, abnormal Papanicolaou tests). We compared proportions of HPV uptake, stratified by age, and geographic region using t-tests and Chi-square tests. We also assessed uptake in non-SID subcohorts of patients with asthma and DM1. We utilized multivariable logistic regression models to compare the odds of uptake of ≥1 vaccine and 3 vaccines between females age 11–26 years with SID and without SID, adjusting for age, geographic region, healthcare utilization, and comorbidities. We excluded females under 11 years of age from this regression analysis in order to focus on the individuals for whom routine use is recommended by the ACIP. We also conducted secondary analyses with multivariable logistic regression models to compare vaccine uptake in the SID cohort to the non-SID subcohort with asthma, and to a non-SID cohort that excludes individuals with asthma or DM1.

All analyses were performed using SAS 9.2 (SAS Institute Inc. Cary, NC). Ethics approval for this study was obtained from the Partners Healthcare Institutional Review Board at Brigham and Women’s Hospital. All patient information was anonymous and de-identified prior to our receipt of the data and therefore informed consent was not possible or required.

## Results

We initially identified 29,255 individuals with SID and 117,020 age, sex and index date matched individuals without SID. The mean age in both cohorts was 18.7 (SD 4.8) and 59.4% were female. Within these cohorts, we restricted our analyses to the 5,643 patients with SID and 20,643 without SID who had ≥24 months of continuous follow-up after their index date. The mean age for both cohorts was 18.1 years (SD 4.9); mean enrollment was 2.3 years (SD 0.9), and 52% of those with SID were female compared to 54% without. This gender difference in the matched cohorts is due to the restriction to include only individuals with ≥24 months of continuous follow-up. During the baseline period, those with SID had significantly greater healthcare utilization, including more frequent outpatient visits, primary care and obstetrics/gynecology visits, compared with those without SID (Table 1). Those with SID also had a higher number of abnormal Papanicolaou tests, STDs, and smoking-related claims.

**Table 1:** Baseline characteristics of the systemic inflammatory diseases (SID) cohort, the non-SID cohort, and the non-SID asthma subcohort

Characteristics*	SID Cohort^+^	Non-SID Cohort	Non-SID Asthma Subcohort
	**N = 5,643**	**N = 20,643**	**N = 1,083**
**Females- N (%)**	2,914 (51.6)	11,150 (54.0)	537 (49.6)
**Age, years—Mean ± SD**	18.1 + 4.9	18.1 + 4.9	17.2 + 5.1
**Abnormal Papanicolaou Tests—N (%)**	79 (2.7)	220 (2.0)	7 (0.65)
**Smoking—N (%)**	113 (2.0)	234 (1.1)	18 (1.7)
**Sexually Transmitted Diseases—N (%)**	469 (8.3)	888 (4.3)	87 (8.0)
**All Outpatient Physician Visits—Mean (SD)**	6.1 (5.0)	2.3 (2.7)	4.9 (4.2)
**Primary Care Visits- Mean (SD)**	3.3 (3.7)	1.5 (2.3)	3.4 (4.7)
**Obstetrics/Gynecology Visits—Mean (SD)**	0.4 (1.6)	0.3 (1.2)	0.3 (1.2)
**Geographic Region**			
Northeast	786 (13.9)	1,972 (9.6)	131 (12.1)
Midwest	1,448 (25.6)	6,105 (29.6)	315 (29.1)
South	2,644 (46.9)	9,594 (46.5)	490 (45.2)
West	765 (13.6)	2,967 (14.4)	147 (13.6)

*All p-values comparing SID and non-SID cohort characteristics <0.05, excluding age, South and West. All p-values comparing SID and non-SID asthma cohort characteristics <0.05 excluding sex, sexually transmitted diseases, primary care visits, obstetrics/gynecology visits, Northeast and South.

+Systemic Inflammatory Diseases (SID include: juvenile idiopathic arthritis, rheumatoid arthritis, systemic lupus erythematosus, psoriasis, psoriatic arthritis, inflammatory bowel disease, vasculitis, multiple sclerosis, juvenile dermatomyositis, systemic sclerosis, ankylosing spondylitis, Goodpasture’s syndrome, and sarcoidosis

We also identified a subcohort of 1,083 non-SID subjects with asthma. The mean age was 17.2 (SD 5.1) and 437 (49.6%) were female. Compared with the SID cohort, the asthma cohort had a similar number of primary care and obstetrics/gynecology visits and STD claims, and fewer smokers and abnormal Papanicolaou tests (Table 1). We also identified a subcohort of 91 subjects with DM1. The mean age was 19 (SD 4.3) and 38 (41.8%) were female. The mean number of outpatient visits for patients with DM1 was 5.4 (SD 3.7).

The disease distribution of patients with SID is presented in Table 2. Psoriasis, psoriatic arthritis and IBD were the most prevalent in our cohort. Among 299 individuals with SLE, 32 (10.7%) received ≥1 HPV vaccine; 7.6% of those with IBD, 14.6% with psoriasis or psoriatic arthritis and 14.7% with juvenile idiopathic arthritis or rheumatoid arthritis, similarly received ≥1 vaccine.

**Table 2:** Disease distribution and HPV vaccine uptake by disease in systemic inflammatory disease (SID) cohort.

Systemic Inflammatory Disease	N (%)	Receipt of ≥1 HPV Vaccine—N (% of those with SID)
	N = 5,643	N = 647
Systemic lupus erythematosus	299 (5.3)	32 (10.7)
Inflammatory Bowel Disease	1,894 (33.6)	143 (7.6)
Psoriasis or Psoriatic Arthritis	2,479 (43.9)	363 (14.6)
Juvenile Idiopathic Arthritis or Rheumatoid Arthritis	468 (8.3)	69 (14.7)
Multiple sclerosis	239 (4.2)	12 (5)
Vasculitis	59 (1.1)	4 (6.8)
Other*	205 (3.6)	24 (11.7)

*Other includes dermatomyositis, systemic sclerosis, ankylosing spondylitis, sarcoidosis and Goodpasture’s syndrome.

SID, non-SID and asthma patients all had low percentages of HPV vaccine uptake overall (Table 3). Among females, beginning in 2007, 20.6% with SID, 23.1% without SID, and 22.9% with asthma, initiated the vaccine series. Comparable percentages of females of all ages in both cohorts initiated the vaccine with the highest percentages of receipt—35.5% (SID), 36.1% (non-SID) and 39.8% (asthma)- in the 12–14 year age group. Percentages of vaccine uptake were similar across all age groups in the SID and asthma cohorts. A slightly higher percentage of non-SID 24–26 year-olds received the vaccine compared to the SID cohort (p = 0.01). Vaccine initiation by region was similar across cohorts with the exception of the Midwest, which had higher uptake among females with asthma (30.3%) compared to those with SID (20.4%, p = 0.01) and in the Northeast where females without SID had higher uptake than those with SID (p = 0.02). Of those females receiving the first vaccine, there was no difference in vaccine completion between SID and non-SID cohorts (p = 0.45) and a slightly lower percentage of completion in the asthma cohort compared to the SID cohort (p = 0.01).

**Table 3:** HPV vaccine uptake among females with systemic inflammatory diseases (SID, A), without SID (B), and without SID with asthma (C).

	(A) SID Cohort	(B) Non-SID Cohort	(C) Non-SID Asthma Subcohort	p-value (A vs. B)	p-value (A vs. C)
	N = 2,914	N = 11,150	N = 537		
**HPV Vaccine Uptake**					
≥1 (Initiators)	601 (20.6)	2,576 (23.1)	123 (22.9)	<0.01	0.23
3 (Completers as % of initiators)	319 (53.1)	1,325 (51.4)	58 (47.2)	0.45	0.01
**HPV Vaccine Initiation by Age in Years- N (% of age group)**					
9–11	110 (25.8)	456 (29.6)	21 (22.6)	0.12	0.47
12–14	163 (35.5)	656 (36.1)	39 (39.8)	0.80	0.41
15–17	134 (26.0)	625 (29.1)	24 (25.8)	0.15	0.99
18–20	96 (19.4)	417 (20.8)	17 (21.0)	0.49	0.74
21–23	62 (13.2)	223 (13.5)	13 (15.5)	0.90	0.60
24–26	36 (6.0)	199 (7.7)	9 (10.2)	0.01	0.21
**HPV Vaccine Initiation by U.S. Region—N (% of region)**					
Northeast	80 (21.3)	280 (27.6)	17 (26.2)	0.02	0.38
Midwest	144 (20.4)	771 (23.7)	46 (30.3)	0.06	0.01
South	279 (19.4)	1,095 (20.8)	46 (19.0)	0.24	0.88
West	98 (24.7)	428 (26.4)	14 (18.0)	0.49	0.20

In a small cohort of 38 females with DM1, 6 (15.8%) received ≥1 vaccine and of these, 1 (16.7%) completed the series. Since the quadrivalent HPV vaccine was approved for use in males in 2009 and recommended for routine use in 2011, given the time frame of our study, we predictably found male vaccine initiation to be extremely low; 46 individuals (1.7%) with SID, 143 (1.5%) without SID, and 11 (2%) with asthma. We also observed no significant differences in vaccine uptake by year between 2007 and 2012. We conducted sensitivity analyses among females to examine vaccine uptake for varying lengths of continuous follow-up after the index date (Table 4). While increased follow-up (≥36 months) did yield slightly higher percentages of uptake, results remained consistent with those obtained for ≥24 months.

**Table 4:** Number and percentage of female HPV vaccine initiators (≥1) and completers (3) by systemic inflammatory disease (SID) status and length of continuous follow-up after the index date.

	Time of Continuous Follow-up After SID or Non-SID Index Date								
	≥ 12 months			≥24 months			≥ 36 months		
	N	Initiators	Completers	N	Initiators	Completers	N	Initiators	Completers
		N (% of total)	N (% of initiators)		N (% of total)	N (% of initiators)		N (% of total)	N (% of initiators)
**SID**	5,552	859 (15.5)	406 (47.3)	2,914	601 (20.6)	319 (53.1)	1,476	378 (25.6)	216 (57.1)
**Non-SID**	22,470	3,978 (17.7)	1803 (43.3)	11,150	2576 (23.1)	1,325 (51.4)	5,508	1,554 (28.2)	868 (55.9)

We constructed multivariable logistic regression models to assess uptake of ≥1 HPV vaccine and completion (3 vaccines) among females age 11–26 years. After adjusting for age, region, comorbidities and outpatient physician visits, the odds of receipt of ≥1 HPV vaccine were lower among those with SID compared to those without (Odds ratio (OR) 0.87, 95% CI 0.77–0.98) (S1 Table). The odds of vaccine completion were not different between the two groups (OR 1.03, 95% CI 0.83–1.26). In our model examining receipt of ≥1 vaccine, we found significantly decreased odds of vaccine receipt in older age groups compared to the youngest age group (11–14 years) and in the South and Midwest compared to the Northeast. Individuals with a history of an abnormal Papanicolaou test had 42% higher odds of receiving ≥1 vaccine compared to those without an abnormal test. There were no significant differences in the predictors in our model examining receipt of 3 HPV vaccines other than reduced odds in the South compared to the Northeast (OR 0.77, 95% CI 0.60–0.98).

In a secondary analysis we compared vaccine uptake of ≥1 and 3 vaccines among females age 11–26 years in the SID cohort and in the non-SID asthma subcohort (S2 Table). Here we found no statistically significant difference in the odds of receipt of ≥1 vaccine (OR 0.88, 95% CI 0.68–1.16) or 3 vaccines (OR 1.25, 95% CI 0.82–1.89) among patients with SID compared to those with asthma. In an additional secondary analysis, we compared uptake of ≥1 and 3 vaccines in the SID cohort to a non-SID cohort that excludes individuals with asthma and DM1. Our results were unchanged from our original model that compared the SID cohort to the overall non-SID cohort (OR of ≥1 vaccine 0.87, 95% CI 0.77–0.98, OR of ≥3 vaccines 1.01, 95% CI 0.81–1.25).

## Discussion

Our study finds strikingly low uptake of the HPV vaccine among individuals with and without SID. Prior U.S.-based studies demonstrate HPV vaccine uptake in females ranging from 11% to 58%.[8,23–26] Overall, our findings of 20.6% in females with SID, 23.1% without SID, and 22.9% with asthma, are in line with these studies. In addition, we found geographic differences in uptake with the lowest odds of vaccine receipt in the South. This has similarly been shown in prior studies.[27,28]

A number of factors likely contribute to the overall low uptake in this nationwide population. When stratified by age, a higher percentage of vaccine initiation (>35%) occurred in females 12–14 years. Lower rates among other age groups may reflect a lack of awareness of approval for ages 9–26, and the ACIP recommendation for routine vaccination of females starting at age 11 and targeting ages 11–12.[7] The health insurer from which these data arose did not fully cover the vaccine for males until 2012, leading to extremely low uptake among males during this study’s timeframe. Additionally, adolescents who are uninsured in the U.S. may have higher HPV vaccine uptake compared to those with commercial insurance coverage because of federal and state programs that subsidize all vaccinations.[29] However, United HealthCare covers all vaccinations recommended by the U.S. ACIP without requiring a visit copayment.

Despite an increased risk of persistent HPV infection and greater healthcare utilization in SID patients, in our adjusted models with found a small but statistically significant reduced odds of HPV vaccine receipt. Comparing SID females to a subcohort of non-SID females with asthma, we found no significant differences in odds of vaccine initiation or completion. We interpret our findings as very low uptake in the entire population overall, among patients with SID, among those without SID, and among those with chronic conditions such as asthma and DM1. Despite the small, statistically significant reduced odds in the SID population, the results are comparable with the non-SID population and with those with asthma. We found this particularly relevant because we hypothesized that we would observe higher uptake among SID patients given their increased vulnerability to persistent HPV infection. One prior study also demonstrated that females with a history of juvenile rheumatoid arthritis or rheumatoid arthritis had comparable rates of HPV vaccine initiation compared to those without.[30]

Our observation of comparable and not heightened HPV vaccine uptake among patients with SID may highlight insufficient access to preventive care services among SID patients, even in the setting of increased visits to primary care providers. Primary care providers may be hesitant to provide vaccinations in general to patients who are immunosuppressed due to concern for an inadequate response, or a fear of precipitating infection or underlying disease flares.[31] This may also hold true for patients with chronic diseases such as asthma explaining the comparable odds of vaccine receipt in this population compared to those with SID. One study showed that chronic diseases in general might be a barrier to breast and cervical cancer screening.[32] Prior studies in SLE patients revealed less preventative services among younger patients and specifically, lower rates of cervical cancer screening compared with the general population.[33,34]

There also may be a lack of primary care provider awareness of the potential increased risk of cervical dysplasia and cervical cancer in SID patients. Subspecialists may provide the majority of primary care services to young patients with SID. The HPV vaccine may not be stored in these clinics, which may explain lower uptake despite increased healthcare use. The shortage of pediatric rheumatologists in the U.S. may also limit the time allotted for each patient and management of the rheumatic disease is prioritized.[35] Providers may also be hesitant to offer a vaccine that is not mandated to chronically ill children to minimize any adverse events. Similarly, parents may seek to avoid subjecting children with chronic conditions to additional interventions that are not required. In addition, rare case reports describing autoimmune disease incidence following HPV vaccine use may have produced uncertainty about vaccine safety.[14]

We also note that the SID cohort had increased percentages of STDs and smoking-related claims compared to the non-SID cohort. The asthma subcohort also had increased percentages of STDs. This may reflect surveillance bias as the SID and asthma cohorts also had a higher average number of outpatient provider visits. Among patients with SID, there may also be an increased awareness of infection risk, particularly among those receiving immunosuppressive medications, leading to increased screening for STDs. A prior study also demonstrated that adolescents with chronic conditions were more likely to engage in high-risk behaviors such as smoking, compared to their healthy peers.[36] This provides further impetus to encourage HPV vaccination among those with SID and with other chronic diseases as well.

This study has limitations. Misclassification of SID diagnoses in the formation of our cohorts may have reduced the contrast between the SID and non-SID enrollees and biased results towards the null. Vaccinations that are paid out-of-pocket may not generate insurance claims and would not be captured in this study, although we anticipate this would be rare given the high cost of the HPV vaccine series (390 U.S. dollars), and the lack of copayment required by this commercial insurance plan. As mentioned, this study was premature to allow for a comprehensive assessment of HPV vaccine uptake in males. In addition, we do not have information about race/ethnicity, which has been shown in prior studies to potentially influence rates of HPV vaccine initiation.[23,37] We were therefore unable to examine this factor in our multivariable analyses, or stratify by race/ethnicity to delineate whether this may contribute to the overall low uptake we observed. Similarly, information on socioeconomic status is not available and we were therefore unable to assess the potential influence of socioeconomic status on vaccine uptake in this SID and non-SID population. However in this insured, primarily employed cohort, we do not suspect that there was a large socioeconomic status gradient that skewed our results. In addition, while claims data provide nationwide records of vaccine uptake that are not influenced by recall bias or volunteer bias, future qualitative studies are needed to understand potentially important motivations behind inadequate vaccine uptake among patients with SID.

## Conclusions

Overall, we demonstrate strikingly low uptake of the HPV vaccine in a nationwide cohort of commercially insured individuals. Patients with SID had modestly reduced odds of HPV vaccine initiation compared to those without SID, and nearly equivalent odds of initiation compared to those with asthma. Despite the demonstrated safety of the vaccine in patients with SID, an increased risk of sustained HPV infection, and greater healthcare utilization, no increase in vaccine uptake was observed. Further public health interventions that target young adults, parents, primary care physicians, and subspecialists, are needed to promote increased HPV vaccination overall and especially among higher risk individuals with SID.

## Supporting Information

S1 Table Multivariable regression models to compare the odds of receipt of ≥1 and 3 HPV vaccinations among females age 11–26 with and without systemic inflammatory diseases (SID). (DOCX)

S2 Table Multivariable regression models to compare the odds of receipt of ≥1 and 3 HPV vaccinations among females age 11–26 with systemic inflammatory diseases (SID) to those without SID with asthma. (DOCX)
