# Supplementary material for: Human Papillomavirus Vaccine Uptake among Individuals with Systemic Inflammatory Diseases
Source: PLoS One. 2015 Feb 18;10(2):e0117620. doi: 10.1371/journal.pone.0117620 (PMC4334247; doi:10.1371/journal.pone.0117620)
Supplement: S1 Table — (DOCX) [file pone.0117620.s001.docx]

| **Covariate** | **>1 HPV Vaccine** | | **3 HPV Vaccines** | |
| --- | --- | --- | --- | --- |
|  | **Odds Ratio (OR)** | **95% Confidence Interval (CI)** | **Odds Ratio**  **(OR )** | **95% Confidence Interval (CI)** |
| **SID (ref: Non-SID)** | 0.87 | 0.77-0.98 | 1.03 | 0.83-1.26 |
| **Age (years)**- [ref=11-14] |  |  |  |  |
| 15-18 years | 0.70 | 0.63-0.77 | 0.98 | 0.82-1.16 |
| 19-22 years | 0.38 | 0.34-0.43 | 0.92 | 0.74-1.13 |
| 23-26 years | 0.19 | 0.17-0.22 | 1.28 | 0.99-1.66 |
| **Geographic region** - [ref=Northeast] |  |  |  |  |
| Midwest | 0.79 | 0.68-0.92 | 0.89 | 0.69-1.16 |
| South | 0.66 | 0.57-0.77 | 0.77 | 0.60-0.98 |
| West | 0.94 | 0.79-1.11 | 0.78 | 0.59-1.04 |
| **Abnormal Papanicolaou Tests** | 1.42 | 1.03-1.95 | 0.98 | 0.55-1.74 |
| **Smoking History** | 0.73 | 0.46-1.18 | 0.69 | 0.28-1.70 |
| **Sexually Transmitted Disease** | 0.99 | 0.82-1.19 | 0.90 | 0.65-1.25 |
| **Number of Outpatient Physician Visits*** | 1.01 | 0.99-1.02 | 1.01 | 0.98-1.03 |
| *Examined as a linear variable | | | | |
