# Supplementary material for: Human Papillomavirus Vaccine Uptake among Individuals with Systemic Inflammatory Diseases
Source: PLoS One. 2015 Feb 18;10(2):e0117620. doi: 10.1371/journal.pone.0117620 (PMC4334247; doi:10.1371/journal.pone.0117620)
Supplement: S2 Table — (DOCX) [file pone.0117620.s002.docx]

| **Covariate** | **>1 HPV Vaccine** | | **3 HPV Vaccines** | |
| --- | --- | --- | --- | --- |
|  | **OR** | **95% CI** | **OR** | **95% CI** |
| **SID (ref: Asthma, non-SID)** | 0.88 | 0.68-1.16 | 1.25 | 0.82-1.89 |
| **Age (years)**- [ref=11-14] |  |  |  |  |
| 15-18 years | 0.64 | 0.52-0.80 | 0.91 | 0.62-1.32 |
| 19-22 years | 0.37 | 0.29-0.48 | 0.85 | 0.55-1.32 |
| 23-26 years | 0.15 | 0.11-0.20 | 1.24 | 0.70-2.19 |
| **Geographic region-** [ref=Northeast] |  |  |  |  |
| Midwest | 0.87 | 0.66-1.26 | 0.98 | 0.58-1.64 |
| South | 0.71 | 0.54-0.93 | 0.86 | 0.53-1.39 |
| West | 1.00 | 0.71-1.41 | 0.80 | 0.45-1.44 |
| **Abnormal Papanicolaou Tests** | 1.71 | 0.94-3.11 | 0.94 | 0.31-2.86 |
| **Smoking History** | 0.78 | 0.36-1.68 | 1.02 | 0.24-4.29 |
| **Sexually Transmitted Disease** | 0.91 | 0.66-1.26 | 0.59 | 0.33-1.04 |
| **Number of Outpatient Physician Visits*** | 1.00 | 0.98-1.02 | 0.99 | 0.97-1.02 |
| *Examined as a linear variable | | | | |
